# Avidity of Anti-Circumsporozoite Antibodies following Vaccination with RTS,S/AS01_E_ in Young Children

**DOI:** 10.1371/journal.pone.0115126

**Published:** 2014-12-15

**Authors:** Ally Olotu, Frederic Clement, Erik Jongert, Johan Vekemans, Patricia Njuguna, Francis M. Ndungu, Kevin Marsh, Geert Leroux-Roels, Philip Bejon

**Affiliations:** 1 KEMRI-Wellcome Trust Research Programme, Centre for Geographic Medicine Research, Coast, Kilifi, Kenya; 2 Centre for Vaccinology, Ghent University, Ghent, Belgium; 3 GlaxoSmithKline Vaccines, Wavre, Belgium; 4 Ifakara Health Institute, Bagamoyo, Tanzania; Queensland Institute of Medical Research, Australia

## Abstract

**Background:**

The nature of protective immune responses elicited by immunization with the candidate malaria vaccine RTS,S is still incompletely understood. Antibody levels correlate with protection against malaria infection, but considerable variation in outcome is unexplained (e.g., children may experience malaria despite high anti-circumsporozoite [CS] titers).

**Methods and Findings:**

We measured the avidity index (AI) of the anti-CS antibodies raised in subgroup of 5–17 month old children in Kenya who were vaccinated with three doses of RTS,S/AS01_E_ between March and August 2007. We evaluated the association between the AI and the subsequent risk of clinical malaria. We selected 19 cases (i.e., with clinical malaria) and 42 controls (i.e., without clinical malaria), matching for anti-CS antibody levels and malaria exposure. We assessed their sera collected 1 month after the third dose of the vaccine, in March 2008 (range 4–10 months after the third vaccine), and at 12 months after the third vaccine dose. The mean AI was 45.2 (95% CI: 42.4 to 48.1), 45.3 (95% CI: 41.4 to 49.1) and 46.2 (95% CI; 43.2 to 49.3) at 1 month, in March 2008 (4–10 months), and at 12 months after the third vaccination, respectively (p = 0.9 by ANOVA test for variation over time). The AI was not associated with protection from clinical malaria (OR = 0.90; 95% CI: 0.49 to 1.66; p = 0.74). The AI was higher in children with high malaria exposure, as measured using the weighted local prevalence of malaria, compared to those with low malaria exposure at 1 month post dose 3 (p = 0.035).

**Conclusion:**

Our data suggest that in RTS,S/AS01_E_-vaccinated children residing in malaria endemic countries, the avidity of anti-circumsporozoite antibodies, as measured using an elution ELISA method, was not associated with protection from clinical malaria. Prior natural malaria exposure might have primed the response to RTS,S/AS01_E_ vaccination.

## Introduction

RTS,S consists of 19 copies of the central tandem repeats and C-terminal region of the *P. falciparum* circumsporozoite protein (CS) fused to hepatitis B surface antigen (HBsAg), and co-expressed with unfused HBsAg in *Saccharomyces cerevisiae cells* [1]. The protein encodes both B and T cell epitopes. The antigen is administered with Adjuvant system (AS01), a liposome-based adjuvant system that contains 3-O-desacyl-4′-monophosphoryl lipid A and the saponin Quillaja saponaria Molina, fraction 21 “(QS21, Antigenics Inc., a wholly owned subsidiary of Agenus Inc., Lexington, MA, USA)”.

RTS,S is highly immunogenic [2], [3], [4], [5], inducing both high titers of CS antibodies (anti-CS) and CS-specific CD4 T cell responses. There is evidence that anti-CS titers correlate with protection in controlled human malaria infection in malaria-naïve adults [2], [6], and natural malaria infection in adults and children in malaria-endemic regions [7], [8]. We have recently demonstrated a non-linear association between anti-CS titers and protection from clinical malaria in children 5–17 months residing in malaria-endemic countries [9], and in children aged 6–10 weeks anti-CS titers were found to be correlated with protection from clinical malaria [10]. However there was no association between anti-CS titers and protection from clinical malaria in children 1–4 years old in Mozambique [11].

Even in trials where correlations between anti-CS titers and outcome were observed, considerable variation in outcome remained unexplained (e.g. children may experience malaria despite high titers). Some studies suggest an association between cell-mediated immune responses (specifically CD4 T cell responses) and protection against clinical malaria in children, albeit of lesser importance than antibody responses [12]. However, substantial variability in protection remains unexplained even after accounting for anti-CS antibody and CD4 T cell responses (i.e. there are unprotected children with high titer antibodies and strong CD4 T cell responses).

In addition to the quantity of antibodies, quality of antibodies may determine protection following vaccination against diverse pathogens [13]. In early phase of adaptive immune response antibody-secreting cells usually produce antibodies of low affinity. These cells then proliferate within germinal centers where somatic hypermutation of V(D)J immunoglobulin gene and antigen-driven selection of high-affinity antibody-producing B cells occurs. Antibody affinity is difficult to measure since it requires monoclonal antibodies, purified antigen, and it must be carried out under strict chemical conditions. However antibody avidity can be used as the surrogate marker for affinity following vaccination. Avidity is defined as the antigen binding capacity resulting from the addition of all epitope-specific affinities of antibodies in a serum [14]. High avidity antibodies appear important in the protection conferred by *Haemophilus influenza* type b vaccine, Hepatitis B vaccine and Pneumococcal conjugate vaccine [15], [16], [17]. The avidity of anti-CS antibody contributes to protection against malaria in a mouse model [18].

To date, no study has investigated the role of avidity of RTS,S-induced anti-CS antibodies in protection against malaria infection among RTS,S vaccinees in the field. Here we report the results of such study in children 5–17 month residing in Kilifi, Kenya who were immunized with RTS,S/AS01_E_.

## Materials and Methodology

### Vaccine and subjects

Serum samples from a phase IIb randomized controlled trial originally designed to determine the efficacy of RTS,S/AS01_E_ against *Plasmodium falciparum* clinical malaria in 5–17 month old children were used (ClinicalTrials.gov number, NCT00380393) [12], [19]. All children received all three doses of RTS,S/AS01_E_ between March and August 2007. The candidate vaccine was given intramuscularly in the right deltoid area in a 0, 1, 2 month schedule. Blood samples were collected at screening, at 1 month after the third dose of vaccine, in March 2008 (range 4–10 months (mean 8 months) post dose 3) and at 12 months after the third dose of vaccine for the assessment of antibodies to *P. falciparum* CS repeat region (anti-CS antibodies).

Informed written consent was obtained from parents of the study participant using approved Swahili or Giriama consent forms. All the parents signed the informed consent and were provided with the copy of informed consent and participant information sheet. Illiterate parents thumb printed the forms with independent literate witness countersigning. The original study was approved by the Kenya Medical Research Institute National Ethics Committee, Western Institution Review Board and Oxford Tropical Research Ethics Committee.

### Study design

A nested case-control study was conducted to investigate the association between vaccine-induced anti-CS antibody avidity and protection from clinical malaria. Cases were defined as children who had at least one episode of clinical malaria (axillary temperature ≥37.5°C and P falciparum parasitaemia >2500/µL) during the 15 months of follow-up beginning 2 weeks after the 3^rd^ dose of vaccine while controls were children who did not experience any clinical malaria episodes.

The study was conducted in villages of Junju and Pingilikani in Kilifi district. The two areas have moderate malaria transmission based on parasite prevalence rates [20]. Malaria exposure was measured as the weighted local prevalence of malaria cases within a 1 km radius of each index child, or “exposure index”, as previously described [21]. Malaria exposure was considered “high” if the exposure index was above the cohort mean and “low” if the exposure index was below the cohort mean.

Due to cost and allowable time to accomplish the study, only a fraction of the available samples could be analyzed. We randomly selected 19 cases and 42 controls from 295 RTS,S/AS01E vaccinees with immunogenicity data matching for the level of peak anti-CS levels and malaria exposure. The selected sample size was enough to provide 80% power to detect an odds ratio of 3 between cases and controls given 50% probability of exposure (antibody avidity with protective effect) among controls and an alpha of 0.05. In an exploratory analysis we compared the avidity indices of controls (protected children) who had low antibody level (in the lower tertile) and high malaria exposure with cases to determine if the avidity index could explain the protection. All selected cases and controls had their samples assessed for the anti-CS avidity at three time points i.e. 1 month, March 2008 (range 4–10 months) and at 12 months post dose three. Avidity was not measured in screening samples because anti-CS titers were undetectable before vaccination.

### Anti-CS antibody titers

Anti-CS antibody titers were determined by standard enzyme-linked immunosorbent assay (ELISA) developed by GSK Biologicals [22]. Plates were adsorbed with the recombinant antigen R32LR, which contains the sequence [NVDP(NANP)_15_]_2_LR and antibody titers were calculated using a reference standard curve and expressed in ELISA units (EU) per mL. A cut-off point for positive titers was 0.5 EU/mL.

### Anti-CS avidity assay

We used a single concentration ammonium thiocyanate (NH_4_SCN) elution ELISA to determine the avidity of the polyclonal anti-CS antibodies. Polystyrene microtiter plates were coated overnight at 4°C with 2.5 µg/ml R32LR protein in coating buffer. Following washing, the plates were blocked with 200 µl PBS-5% skim milk per well for 1 hour at 25°C in a horizontal orbital shaker (Skatron 300).

We conducted 8-fold serial dilutions in duplicate. A pre-dilution at 1∶100 was done for all serum samples with anti-CS titer above 200 EU/mL before the beginning of serial dilutions. Serum samples with anti-CS titer below 200 EU/mL were not pre-diluted. Standard (serum from malaria naïve adult vaccinated with R32LR attributed an arbitrary value of 109 EU/mL provided by GSK Vaccines), negative control (i.e. anti-CS negative serum from malaria naïve adult provided by GSK Vaccines) and positive control (i.e. a pool of anti-CS human sera approximately at 100 EU/ml that demonstrated an AI of 43.1±3.8, provided by GSK Vaccines) were added in each plate and serially diluted like samples. The plates were then incubated for 2 hours at 37°C.

Each serum sample was processed in two different plates; one treated with NH_4_SCN, and one untreated plate. Both plates were washed twice with 0.05% Tween-20 in PBS (wash buffer). A 1 M solution of NH_4_SCN in laboratory grade water was added in the treatment plate while 0.05% Tween-20 in PBS was added in the control plate and both were incubated for 30 min at 25°C. The plates were then washed three times with wash buffer. After a third wash, anti-human IgG conjugated to horseradish peroxidase (HRP) was added and incubated for 30 min at 25°C before washing. After 30 min incubation with chromogen substrate (3,3′,5,5 Tetramethylbenzidine [TMB] and H_2_O_2_) at 25°C (yielding a blue color), the reaction was stopped with 50 µl of 1 N sulphuric acid changing the color to yellow. The intensity of the color was proportional to the titer of the anti-CS IgG antibodies contained in the sample. Absorbance at 450 nm was read by use of an automatic microtiter plate reader. Samples Optical densities (OD’s) were converted into EU/ml using a standard curve at the linear segment of the curve and mean values used to estimate avidity index. Linearity of the assay was demonstrated by the observation that for a high concentration sample, serially diluted sample was able to reproduce the same Avidity Index (AI) over the entire anti-CS analytical range. The AI is defined as the ratio of the quantity of anti-CS antibodies (in EU/ml) that remained bound to the coated antigen after treatment with NH_4_SCN divided by the quantity of antibodies (in EU/ml) that remained bound to the coated antigen in the control plate. The AI for each serum represents the composite value across all the dilutions.

### TNF-α-producing CD4 T-cells immune response

Whole-blood intracellular staining assay was used to determine the frequency of CD4 T cells producing TNF-α as previous described [12]. Stimulation of blood was done within 3 hours after blood withdrawal and samples were stored 3 to 4 months before staining.

### Statistical Analysis

Anti-CS avidity measures were not transformed in the analysis and were presented as percentage (by multiplying the ratio by 100). The anti-CS titers and avidity indices were presented for each group as arithmetic mean±95% confidence interval per group. Students T-test was used to compare the avidity indices and antibody titers between groups. A one-way analysis of variance (ANOVA) was used to test for the difference in antibody titers and antibody avidity during follow up (1 month, ∼8 and 12 months post dose three). Unconditional logistic regression analysis was used to estimate odds ratios (ORs) and 95% Confidence intervals adjusting for TNFα-producing CD4 T cells, bed net use and malaria exposure (high or low). Cluster sandwich covariance matrix estimator was used to account for the multiple entries per child. No adjustment was made for the variable (i.e. anti-CS titers) used to select cases and controls to avoid bias. All analyses were done in STATA (version 12; Stata Corp).

## Results

All children received all three doses of RTS,S/AS01_E_ between March and August 2007.

A total of 162 sera (50 sera obtained 1 month post dose 3, 51 sera obtained on March 2008 (range; 4–10 months post dose 3) and 61 sera obtained 12 month post dose 3) from 61 study participants were available for the analysis.

**Figure 1:**
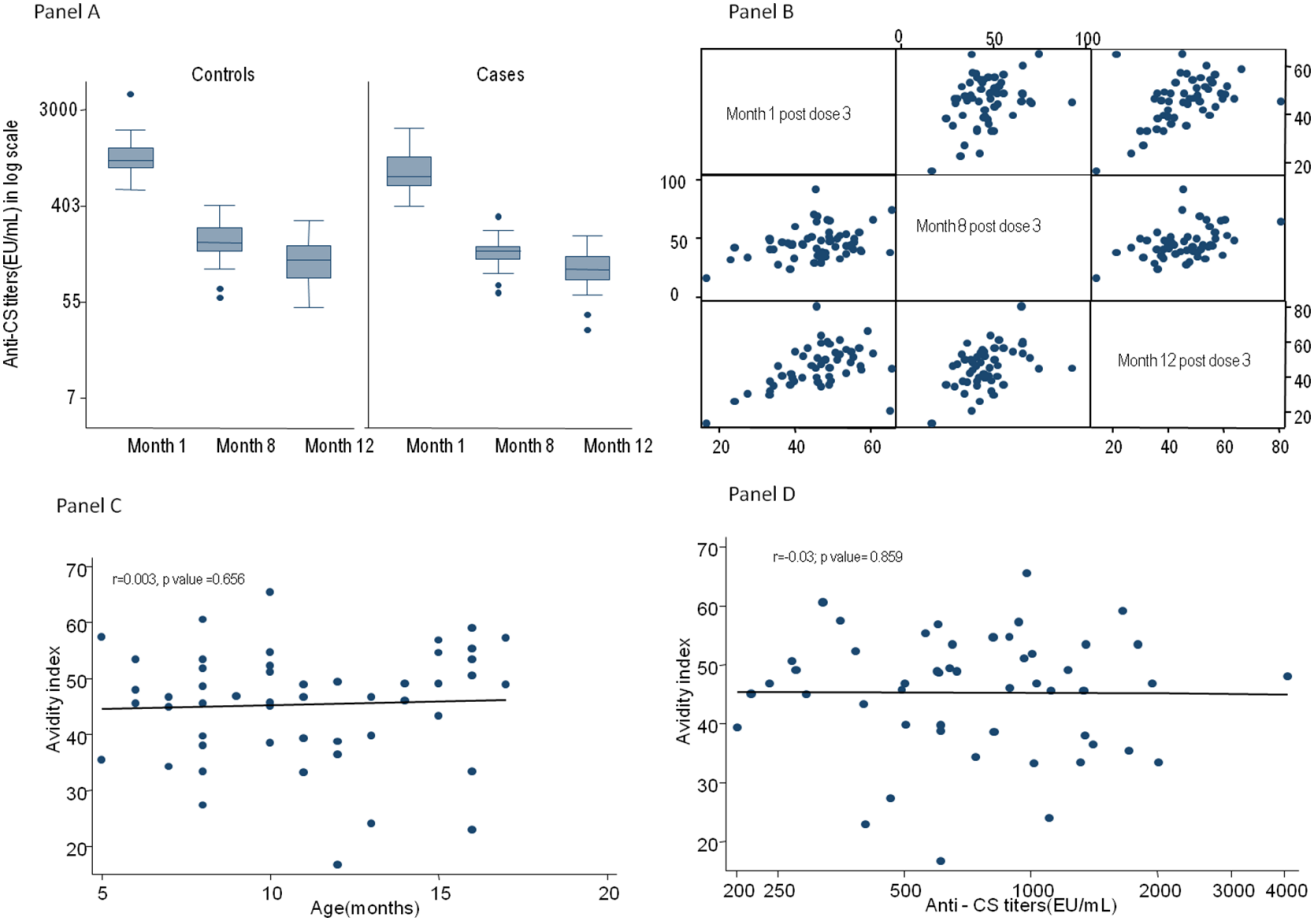
Panel A: Anti-CS titers in cases, controls and 19 protected children with low anti-CS antibodies titer and high malaria exposure index during 15 months of follow-up. Panel B: Matrix diagram showing correlation between antibody avidity measured at three time points during the follow up. Significant correlation observed between AI at 1 month post-dose 3 and at ∼8 months post-dose 3 (r = 0.33, p value = 0.0111) and at 1 month and 12 months post-dose 3 (r = 0.48, p value = 0.0002). Panel C: Correlation between anti-CS antibody avidity at 1 month post dose 3 and age (r = 0.003, p value = 0.656) Panel D: Correlation between anti-CS antibody avidity and anti-CS antibody titers at 1 month post dose 3 (r = –0.03; p value = 0.859).

The geometric mean titer of anti-CS titer in a subset of children in this study 1 month after the third vaccination was 732.1 EU/mL (95% CI, 608.4 to 880.8) and this fell to 105.3 EU/mL (95% CI, 83.8 to 132.3) and 64.7 EU/mL (95% CI, 50.4 to 83.1) at 6.5 month and 12 month post vaccination 3 respectively (F test = 125 p<0.001 by ANOVA test). The dynamics of anti-CS titers in cases and controls is shown in Fig. 1 (Panel A).

### Anti-CS avidity

The arithmetic mean AI of polyclonal anti-CS antibodies at 1 month, March 2008 (∼8 months) and 12 months post dose 3 was 45.2 (95% CI: 42.4 to 48.1), 45.3 (95% CI: 41.4 to 49.1) and 46.2 (95% CI; 43.2 to 49.3), respectively (Table 1). The AI did not differ between the three time points (F test = 0.12 p = 0.9 by ANOVA test).

**Table 1:** Avidity indices in cases, controls and a subset of 19 controls with high exposure rates and low anti-CS titers at different sampling times.

Sampling time Monthsafter 3^rd^ vaccine dose	Cases Avidityindex (95% CI)	Controls Avidity index (95% CI)		P value*
		All	High exposureand low anti-CS titer	
1	47.1(42.8–51.4)	44.6(38.6–50.6)	44.4(39.4–49.4)	0.418
∼8	48.4(43.8–52.9)	42.2(33.9–50.4)	45.2(37.5–52.9)	0.254
12	48(43–53)	44.7(39.2–50.1)	46.3(40.1–52.4)	0.423

*CI: Confidence Intervals.*

*p value for the comparison between cases and controls (all).

Correlations were observed between the AI at 1 month post-dose 3 and at ∼8 months post-dose 3 (r = 0.33, p = 0.0111), as well as at 1 month and 12 months post-dose 3 (r = 0.48, p = 0.0002) (Fig. 1, Panel B).

There was no correlation between the AI and anti-CS titers at all three time points (r = –0.03; p = 0.859, r = 0.05; p = 0.704 and r = 0.16; p = 0.220) or between the AI and age at vaccination (r = 0.003, p = 0.656) (Fig. 1, Panel C and Panel D respectively).

### Avidity and protection from clinical malaria

The AI in cases and controls at 1, ∼8 and 12 months post dose three is shown in Table 1. The AI at all time points was not different from those recorded in cases and controls (F test = 2.69, p = 0.103 by ANOVA) (Table 1). In an exploratory analysis, we showed that avidity indexes indices among controls (i.e. children without episodes of malaria or the “protected group”) were similar to cases (i.e. children with malaria) (Table 1).

The unadjusted odds ratio for clinical malaria was 1.36 (95% CI: 0.89 to 2.1; p = 0.165) for each 10% increase in avidity index, indicating that we had power to exclude an OR of <0.89. Multivariable logistic regression showed no association between the avidity index and protection from clinical malaria (Table 2). TNF-alpha producing CD4 T cells were independently associated with decreased risk of malaria (OR = 0.63, p value = 0.027) while malaria exposure was associated with increased risk for clinical malaria (OR = 11.24, p value = 0.005).

**Table 2:** Multivariable logistic regression analysis for the effect avidity on clinical malaria.

Variables	Odds Ratio (95% CI)	P value
Avidity (per 10% increase in avidity).	0.90 (0.49–1.66)	0.744
CD4^+^-TNF-α cells(per 10-fold increase in frequency)	0.63 (0.41–0.95)	**0.027**
Bed net	1.39 (0.42–4.58)	0.588
Malaria exposure	11.24 (2.08–60.68)	**0.005**

### Avidity and malaria exposure

**Figure 2:**
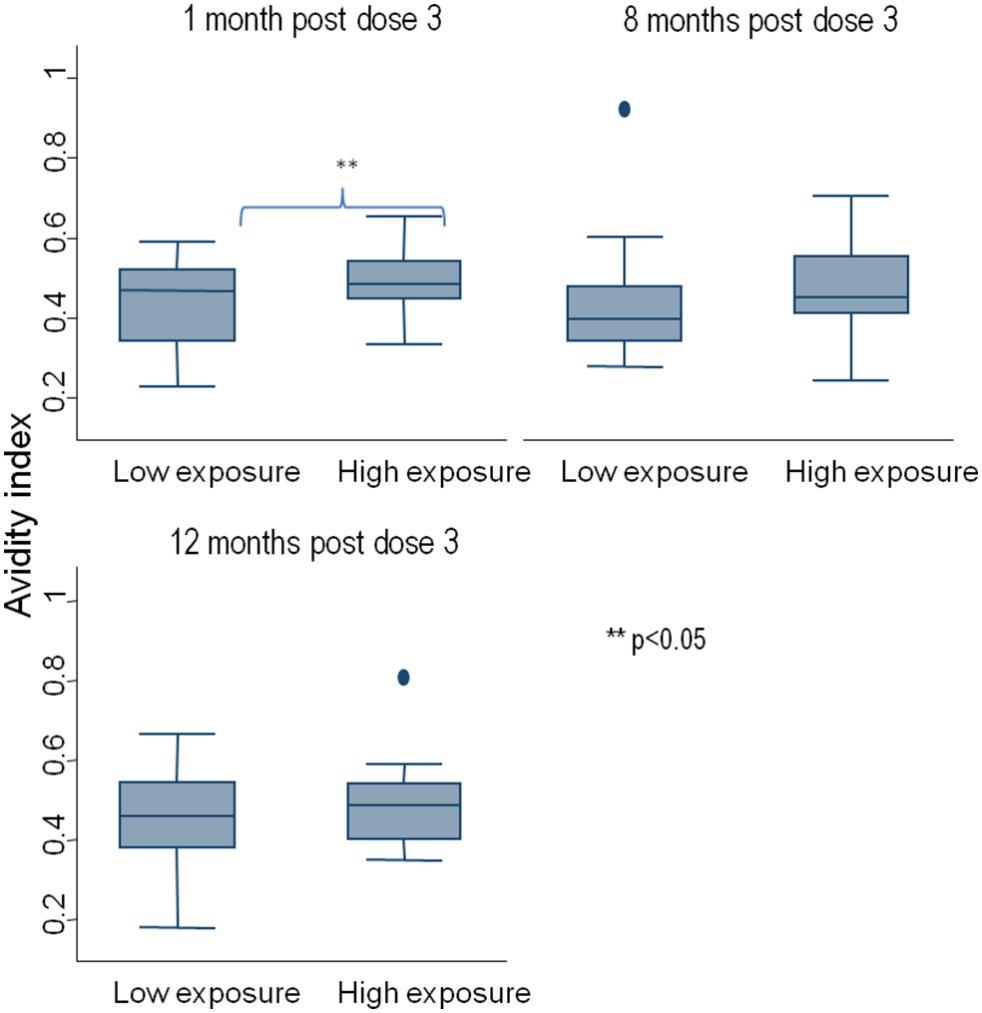
Box plot of anti-CS antibody avidity by level of malaria exposure (based on malaria exposure index) at 1, ∼8 and 12 months post dose 3.

The AI at 1 month post dose 3 was higher in children under high malaria exposure conditions compared to those under low malaria exposure with borderline statistical significance (48.6%, 95% CI: 45.4 to 51.8, versus 42.8%, 95% CI 38.2 to 47.4, p = 0.035 by Students T test) (Fig. 2). This difference was also observed at ∼8 months; 47.9% (95% CI: 42.4 to 53.5) versus 43.2% (37.6 to 48.8) p = 0.2167 and at 12 months; 48.2% (95% CI: 44.3 to 52.1) versus 45.3% (95% CI: 40.6 to 49.9) p = 0.3361 post dose 3 but these were not statistically significant.

## Discussion

The nature of protective immune responses elicited by immunization with RTS,S is still incompletely understood. Although RTS,S-induced humoral and cell mediated immune responses correlate with protection against both asymptomatic and symptomatic parasitaemia [2], [9], [23], there is substantial variation in the rate of protection within individuals displaying similar level of immune responses. Here, we show that antibody avidity is not associated with protection from clinical malaria among RTS,S/AS01_E_ vaccinees with similar levels of anti-CS antibodies aged 5–17 months and residing in a malaria endemic country.

There was no evidence of anti-CS avidity maturation beyond one month post dose 3. Avidity maturation follows B cells activation in a CD4 T cell dependent manner and is the hallmark of immunologic memory [13]. However there is substantial variability in the capacity of vaccines to evoke avidity maturation [24]. Following Hepatitis B vaccination no avidity maturation was observed beyond the third dose although significant increase in avidity occurred between the first and the third dose [15]. Avidity maturation persisted between the third and fourth dose of meningococcal serogroup C (MCC) conjugated vaccine, but there was no change in antibody avidity beyond the fourth dose [25]. In our setting, the avidity maturation process may have been complete by 1 month post dose three. Since avidity maturation has only been evaluated after the third dose, no information is available of its evolution following the preceding doses. Factors such as the type of method and chaotropic agent used, chaotropic agent concentration, chaotropic incubation time and temperature of the reaction [26] can influence the avidity results making comparison across the studies and methods difficult. Avidity index of antibodies to GMZ2 (a hybrid protein consisting of the N-terminal region of the glutamate-rich protein fused in frame to the C-terminal region of merozoite surface protein 3) after three doses was 35% (95% CI 30% to 42%), slightly lower than what we observed for RTS,S induced antibodies. Similarly, the avidity of naturally acquired antibodies to AMA-1 and MSP-1 antigens in children residing in malaria endemic country were considerably lower (median of 13% and 15% respectively) compared to RTS,S induced antibodies to CSP [27]. However both studies used different chaotropic agents (diethylamine and guanidine hydrochloride, respectively) and incubation times (15 and 10 minutes respectively). Potent novel adjuvant (AS01) co-administered with the vaccine and the virus-like particle nature of the RTS,S may explain the high avidity observed in comparison with other studies [28], [29].

We found no association between avidity and protection from clinical malaria. Data in one animal model (*P. berghei*) suggests that induction of sterile immune responses by CSP-based subunit vaccines depends on inducing antibody of the appropriate isotype, specificity and avidity [30]. A study in another animal model (*P. yoelii*) found protection against sporozoite challenge to be independent of antibody avidity and/or isotype [31]. In contrast high affinity antibodies to Plasmodium falciparum merozoite antigens measured by surface plasmon resonance technology have been shown to be protective [32]. One possible explanation for lack of correlation between avidity and protection in the present study may be that the majority of the antibodies induced by three doses of RTS,S/AS01_E_ had exceeded a minimum avidity threshold required for protection and hence we could not detect any variation in outcome. For instance, Bachmann et al. observed lack of correlation between antibody avidity and protection against vesicular stomatitis virus (VSV) once the antibody avidity had reached a minimum threshold [33]. The AI was significantly higher in children with high malaria exposure compared to those with low malaria exposure. In theory, this could be due to progressive anti-CS avidity maturation as a consequence of natural infection and boosting by repeated malaria exposure early in life (prior to vaccination). However, there is no evidence of boosting of RTS,S-induced immune responses by natural exposure in previous studies, and the avidity did not increase over the duration of the study. In fact we found no evidence of avidity maturation of anti-merozoite antibodies with increasing natural malaria exposure in individuals residing in the study area [27]. Alternatively, prior exposure to malaria (as measured using our exposure index) may have primed children to respond with higher avidity antibodies on vaccination [34]. Thus, those with higher exposure may have had higher frequencies of naturally acquired anti-CS memory B cells with higher receptor affinities than those with low exposure. In keeping with this suggestion is the finding that the correlation between AI and malaria exposure was only significant at 1 month post dose 3 and not at subsequent time points. The trial provided insecticide treated bed nets to all study participants at the start of the trial. Although the use of the bed net declined over time, it remained above 70% and we did not find association between bed net use and protection from malaria.

Although avidity was associated with exposure to malaria, there was no indication that avidity might be protective in our case control analysis either with or without adjusting for exposure (Table 2). Among the group of vaccinated children who remained free of clinical malaria episodes despite low antibody titers and apparent exposure to malaria as determined by the exposure index, the avidities were no higher than the randomly selected cases and controls (Table 1). Taken together with our case control analysis (in which we confirmed a previously identified association between protection and cellular immunity) [12], [19], our results suggest that avidity, as measured using an elution ELISA method, does not play a role in determining outcome in the field. Studies where vaccinees are exposed to controlled human malaria infection suggest that antibody levels are more strongly associated with protection than cellular immunity [35].

We used an elution assay with single thiocyanate concentration and serially diluted sera. The method has been extensively used to measure the avidity of various vaccine antigens [14], [24], [36]. The method is devoid of many limitations encountered by an elution method using multiple concentrations of thiocyanate with single sera dilutions. Such limitations include discrepancies in OD results at lower thiocyanate concentration and inability to take into account the effect of serum concentration on the binding of antigen [36]. Surface Plasmon resonance (SPR) is a new technique which has recently been used to measure the avidity of malaria specific antibodies [32]. It measures the binding capacity of an antibody under flow, mimicking the in vivo environment [37]. Studies have shown that the Surface Plasmon resonance does not necessarily correlate with results from thiocyanate avidity assays [32] and therefore further investigation using this new technique may be justified.

We did not observe any correlation between age of the child and antibody avidity or between antibody avidity and antibody levels. Study evaluating the affinity of naturally acquired blood stage antigens using SPR and elution ELISA found positive correlation between antibody levels and its affinity for high but not low affinity antibodies [32]. Despite declining anti-CS titers, antibody avidity was sustained throughout the 15 months of follow-up. Although quantity of antibody may reflect the immediate response to a vaccine, quality of antibody response may be more important in determining the immune status months after vaccination. However Bachmann et al noticed that low avidity antibodies required very high concentration to achieve desired effectiveness [33], which may suggest that increasing and maintaining high antibody titres could be an important strategy to improve and sustain the efficacy of RTS,S.

TNF-alpha producing CD4 T cell responses were independently associated with protection. There was no interaction between avidity and TNF-alpha producing T cells (odd ratio for interaction term = 0.77, p value = 0.319). However CD4 T cells are known to play an important role in antibody affinity maturation through their interactions with cognate B-cells [38].

There are other qualitative aspects of the antibody responses that we did not examine; such as the IgG isotypes in the children and ability of antibodies to inhibit hepatocyte infection in functional assays. IgG3 and IgG1 are the most effective isotypes at mediating antibody-dependent cellular mechanisms like phagocytosis and complement fixation against blood stage malaria antigens, and have been associated with protection from clinical malaria in the field [39], [40], [41]. On the other hand IgG2 may compete with IgG1 and IgG3 and interfere with their activity [42]. Thus, similarly in the case of RTS,S immunization the isotype distribution of the induced response may determine the mechanism by which sporozoites may be taken up and destroyed by monocytes and macrophages [43]. Although the anti-CS antibody response induced by RTS,S in naïve adults is skewed towards IgG1 and IgG2 [44], the isotype distribution in children is unknown. It is also worth noting that our analysis focused on the antibodies to the central repeat region of the RTS,S and not antibodies to the C- terminal flanking region. This potentially excludes some relevant antibody responses which could explain the effectiveness of the vaccine [45]. However, antibodies to repeat CS have been shown to neutralize the infectivity of the sporozoite both in vivo and in vitro [46], [47], [48] and CS repeat region is regarded as the major immunodominant epitope [49], [50].

Our assays measured average avidity of specific polyclonal serum antibodies which comprise of a mixture of antibody subpopulations with different avidities. Avidity indices can vary considerably with each sample and this can only be well depicted by avidity distribution. We cannot comment on this possibility given that we did not store the data in a manner to enable the performance of this analysis.

In conclusion, antibody avidity did not predict protection in RTS,S/AS01_E_ vaccinees with similar levels of anti-CS titers Antibody avidity was higher in children with high malaria exposure suggesting a possible priming effect of natural infection on the RTS,S-induced response.
